# A Modular 3-Degrees-of-Freedom Force Sensor for Robot-Assisted Minimally Invasive Surgery Research

**DOI:** 10.3390/s23115230

**Published:** 2023-05-31

**Authors:** Zonghe Chua, Allison M. Okamura

**Affiliations:** 1 Department of Electrical, Computer and Systems Engineering, Case Western Reserve University, 10900 Euclid Avenue, Glennan Building 514A, Cleveland, OH 44106, USA; 2 Department of Mechanical Engineering, Stanford University, Stanford, CA 94305, USA; aokamura@stanford.edu

**Keywords:** force sensing, minimally invasive surgical robotics, medical robotics

## Abstract

Effective force modulation during tissue manipulation is important for ensuring safe, robot-assisted, minimally invasive surgery (RMIS). Strict requirements for in vivo applications have led to prior sensor designs that trade off ease of manufacture and integration against force measurement accuracy along the tool axis. Due to this trade-off, there are no commercial, off-the-shelf, 3-degrees-of-freedom (3DoF) force sensors for RMIS available to researchers. This makes it challenging to develop new approaches to indirect sensing and haptic feedback for bimanual telesurgical manipulation. We present a modular 3DoF force sensor that integrates easily with an existing RMIS tool. We achieve this by relaxing biocompatibility and sterilizability requirements and by using commercial load cells and common electromechanical fabrication techniques. The sensor has a range of ±5 N axially and ±3 N laterally with errors of below 0.15 N and maximum errors below 11% of the sensing range in all directions. During telemanipulation, a pair of jaw-mounted sensors achieved average errors below 0.15 N in all directions. It achieved an average grip force error of 0.156 N. The sensor is for bimanual haptic feedback and robotic force control in delicate tissue telemanipulation. As an open-source design, the sensors can be adapted to suit other non-RMIS robotic applications.

## 1. Introduction

Respect for tissue [1] or force sensitivity [2], is considered an important skill for performing safe surgery and requires good control of applied forces. Thus, knowledge of the force exerted by a robotic system on the surgical environment is important during robot-assisted, minimally invasive surgery (RMIS) to enable safe tissue handling. Force information can be used to provide haptic feedback to the surgeon, automatically and objectively evaluate their force sensitivity for training and credentialing purposes, and inform the decisions and movements of an autonomous agent.

Force information has been difficult to obtain for the above purposes in RMIS because there is no native distal force sensing in commercial RMIS systems. This is due in part to designers needing to meet the strict requirements for biocompatibility and sterilizability of RMIS instruments while ensuring cost-effectiveness [3,4,5]. Researchers have explored many approaches to developing force sensors that attempt to address the above constraints, with features that allow the overall sensing setup to be biocompatible, sterilizable, and miniature in size. However, none have gained commercial adoption. Furthermore, many designs contain complex electromechanical components that require specialized knowledge to manufacture, assemble, or integrate. This limits their adoption even in the research community.

**Figure 1:**
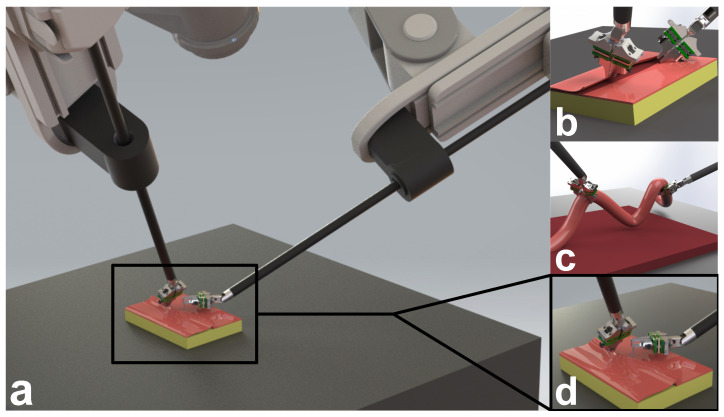
Concept renderings of the 3DoF force sensor design in example use cases requiring bimanual manipulation. (**a**) View of sensorized patient-side manipulators during suturing. Close-up of sensorized forceps during (**b**) blunt dissection, (**c**) running the bowel, and (**d**) suturing.

To make up for the lack of feasible force sensing options for RMIS tools, researchers investigating methods for surgical skill evaluation have often relied on existing, general-purpose, commercially available force sensors like those from ATI Industrial Automation (Apex, NC, USA) that are placed in or under the artificial tissue being manipulated. In such a setup, researchers are limited to RMIS studies using only a single-end effector [6,7] or measuring a single force value for both-end effectors [8,9]. This approach prevents the study of bimanual force-critical tasks such as those shown in Figure 1 and thus limits applicability to real surgery.

One approach that has been identified as a promising potential solution that circumvents the need for end effector force sensors is “contactless” force sensing [5]. This has been explored using physics-based [10,11] or neural network models [12] of the robot to predict joint torques and using vision-based finite element [13] or deep learning methods [14,15,16,17,18,19]. However, these approaches need to be trained or benchmarked against a ground truth. To achieve this, researchers have often used a single environmental force sensor like the ATI sensors noted above. For methods that rely entirely on the robot’s internal state, this approach is feasible because each end effector can be trained separately. However, for methods that rely on measuring environmental changes, such as vision-based methods, this approach has limited applicability to bimanual manipulations where internal ground truth force data cannot easily be resolved.

In this work, we present the design and characterization of a 3-degrees-of-freedom (3DoF) force sensor for RMIS research. The novelty of this sensor is that, compared to jaw-based research sensors, it is easy to manufacture and integrates readily with existing hardware. Compared to prior shaft-mounted sensor designs, it achieves comprehensive lateral, axial, and grip force sensing, making it suitable for a wide variety of research use cases. Compared to commercial off-the-shelf sensors that are mounted to the external environment, our sensor enables bimanual as well as grip force sensing. We achieve this by relaxing the strict constraints on size, biocompatibility, and sterilizability that are necessary for clinical sensors to satisfy, but are less critical for benchtop research applications. To capitalize on its manufacturability and modularity, we have open-sourced the design to enable researchers to adapt the sensor for their desired application both within RMIS and for other robotic applications where deploying commercial sensors is difficult due to size, cost, and customizability requirements.

## 2. Background

Previous works have placed sensors at different locations on the RMIS instrument. These include the jaws, wrist, lower shaft, trocar, upper shaft, and the instrument base. These works have also employed various types of sensing technology, with metal strain gauges, capacitive sensors, fiber Bragg gratings, and infrared (IR) light intensity measurement being the most common technologies employed.

Force sensors located at the jaw have been implemented as both 2DoF [20] and 3DoF sensors [21] using strain gauges and custom jaw flexures. Custom jaw flexures were also employed in [22] for 3DoF force and 2DoF moment sensing using capacitive elements. Such jaw flexures allow forces to be sensed when any part of the tool tip interacts with the environment. This is important during tasks such as blunt dissection (Figure 1b) and running the bowel (Figure 1c), where the tip and back of the tool tip are used for manipulation. This is in contrast to approaches that place sensors on the grasping surface of each jaw and thus only allow forces to be sensed when the environment is grasped [23,24]. By locating the sensors at the jaw, grip force can also be computed from the force measurements at each jaw.

Locating force sensors above the articulated tool wrist reduces the electromechanical integration complexity typical of sensors located at the jaw. Li et al. [25] used a Stewart platform with strain gauges to measure 3DoF forces at the articulated wrist of a custom RMIS tool, while Lee et al. [26] measured 6DoF forces and moments at the articulated wrist using capacitive sensors. Torque sensors were also embedded in the drive pulleys and used to both measure grip force and compensate for noise in the wrist force sensors due to drive cable actuation. Sensors have also been located on the lower shaft of RMIS tools. Shazada et al. [27] and Du et al. [28] used fiber Bragg gratings to measure 2DoF lateral forces. Machaca et al. [29] measured forces using custom strain gauge films for wrist-based haptic feedback, and Wee et al. [30] adopted a similar sensing approach for manual laparoscopic instruments. For Wee et al. [30], the performance of the sensor was only reported for lateral bending.

Sensors have also been placed at the interface of the patient’s body (at the trocar), or outside the body, for example, on the upper shaft or instrument base. Kim et al. [31] used strain gauges at the trocar to measure 2DoF lateral forces, while Fontanelli et al. [32] used IR intensity measurement to do so. At the upper shaft, Hosseinabadi et al. [33] used IR intensity to measure 3DoF forces and moments, although for the three directions of force, precision metrics were reported for only the two lateral directions of force. At the instrument base and upper shaft, Novoseltseva [34] used strain gauges to measure 3DoF forces, with force measurements along the main axis of the tool showing poorer accuracy relative to those along the lateral directions.

## 3. Methods

### 3.1. Target Design Requirements

To realize a force sensor that is accessible to the research community, the sensor design should be easily manufacturable and integrated into existing RMIS tools. This makes sensors located on the upper shaft of the instrument base particularly suitable [4]. However, these designs typically lack accuracy along the main axis of the RMIS tool. Furthermore, they are unable to measure grip force, which can be useful for evaluating surgical skill or for providing feedback to improve tissue manipulation.

RMIS research is often performed with ex vivo or dry lab tasks. This relaxes the requirements on biocompatibility, sterilizability, and size. Thus, placing sensors at the tool jaws does not require complex jaw designs and can use small-size commercial load cells. At the same time, locating the sensor at the jaw reduces measurement noise and enables more accurate measurement of force along the main axis of the tool. Additionally, jaw sensor placement allows for straightforward grip force measurement.

Based on these considerations, we designed a jaw-mounted sensor that can be customized to suit different RMIS tools and different use cases. Our target use case of tissue manipulation requires that all parts of the jaw be able to sense force, and thus, unlike in Kim et al. [23] and Dai et al. [24], the sensing elements cannot be solely mounted on the grasping surfaces of each jaw.

Tissue manipulation forces can be up to 3.8 N in the lateral direction, −10.3 N in the axial compression direction, and 7.8 N in the axial retraction direction [35]. However, there is a need to balance these requirements against the current capabilities of small-size commercial load cells. Based on these considerations, our sensor target force ranges are ±3 N in the lateral direction and ±5 N in the axial direction. In terms of accuracy requirements, the average kinesthetic force difference just noticeable for the human hand is 12.5% [36], and thus the sensor requires a minimum sensor accuracy of 0.375 N in all directions for error imperceptibility.

### 3.2. Electromechanical Design

**Figure 2:**
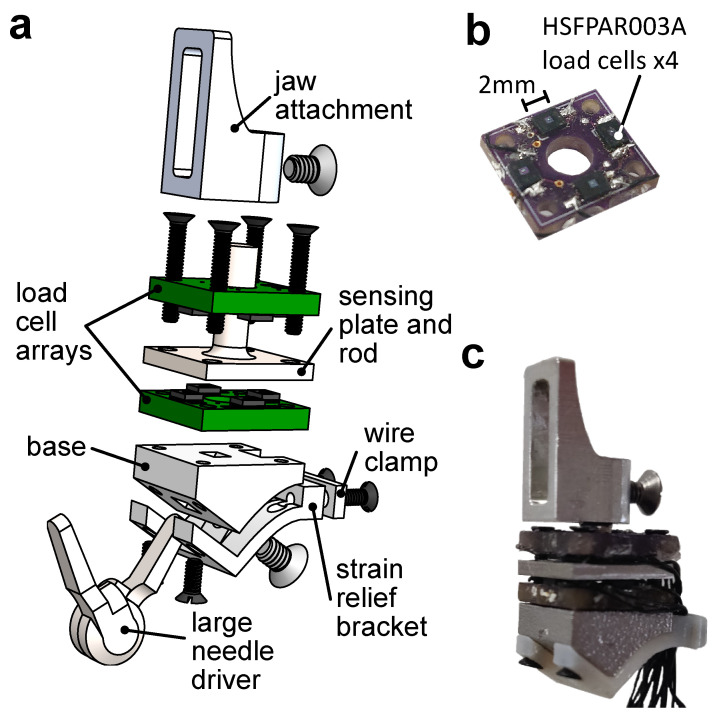
(**a**) Exploded view of the force sensor mounted to one jaw of the da Vinci large needle driver. (**b**) Arrangement of the load cells on the PCB of the load cell array. (**c**) Fully assembled force sensor.

Based on the above design requirements, we designed a 3-degrees-of-freedom force sensor located at the tool jaws. As shown in Figure 2a, the sensor comprises five main parts: (1) the base, (2) bottom load cell array, (3) top load cell array, (4) the sensing plate and rod, and (5) the jaw attachment. An optional strain relief bracket (also shown in Figure 2a) can be added to help secure and route wires.

The base is 3D-printed in 6061 aluminum, and its geometry can be modified to interface with different RMIS tool jaws. In this paper, we present a design that interfaces with the da Vinci Surgical System (Intuitive Surgical, Inc., Sunnyvale, CA, USA) large needle driver jaw using an M2 × 3 set screw. One of two load cell arrays is placed above the top surface of the base and is electrically isolated using Kapton tape.

Each load cell array is a 9.5 × 8.5 × 1.6 mm, 2-layer, FR4-printed circuit board with 4 HSFPAR003A load cells (Alps Alpine, Tokyo, Japan) soldered along the perimeter (Figure 2b). The load cells measure compression forces of up to 8 N and rely on a piezoresistive full Wheatstone bridge, which allows for good temperature stability. The bridge outputs are amplified using AD623 (Analog Devices, Norwood, MA, USA) instrumentation amplifiers with a gain of 21. This results in a sensor response of 3.063 $N\;V^{-1}$. The amplified analog signals from each sensor were recorded on a PC using an Arduino Mega with serial communication at 125 Hz.

The sensing plate and rod were machined out of 303 stainless steel for high stiffness. A second load cell array was placed on the top face of the plate in opposition to the first load cell array. The 2 load cell arrays and the sensing rod and plate were attached to the base using 4 M1.2 × 8 mm screws.

A jaw attachment, which replaces the original tool jaws for grasping, was machined out of 6061 aluminum and was attached to the sensing rod by an M2 × 3 mm set screw. The jaw attachment is interchangeable, allowing for researchers to machine different shapes to suit the task they are studying. Here, we fabricated a generic shape for tissue retraction and palpation that had a height of 12 mm. During manufacturing of the load cell arrays, there are small deviations in the heights of each load cell after soldering. Thus, we enabled consistent contact between the sensing plate and the individual load cells on each side by inserting metal shims. The sensors were preloaded up to a maximum of 1.5 N. The fully assembled sensor is shown in Figure 2c with overall dimensions of 9.5 × 8.5 × 23.8 mm and a weight of 3.33 g. A video of the assembly process is provided in the Supplementary Material (Video S2).

### 3.3. Sensing Principle

**Figure 3:**
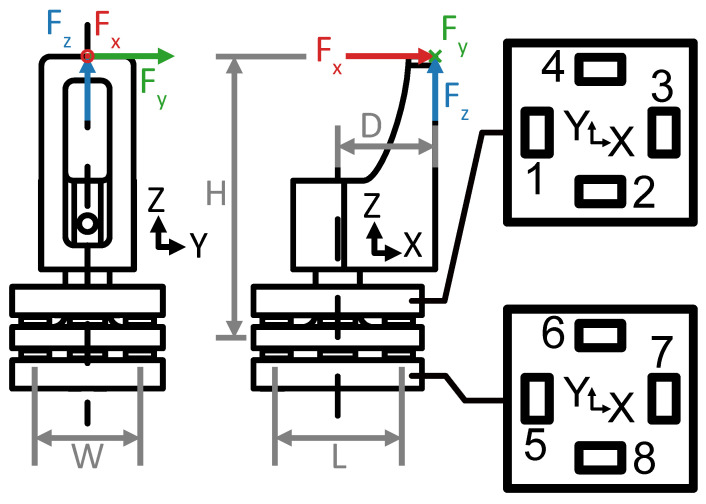
Front and side views of the force sensor with top views of the load cell arrays. Load cells are numbered 1 through 8 corresponding to Equations (1)–(3).

The sensing principle relies on the moment balance about the lateral axes of the device, henceforth referred to as the sensor x- and y-axis, and force balance in the main axis, henceforth referred to as the sensor z-axis. Assuming an interaction at the tip of the jaw attachment, and neglecting the contribution of shear forces to the moment balance, the resulting force and moment equations are

(1) $$M_{x}=F_{y}H-\frac{Lc}{2}\left(v_{2}+v_{6}-v_{4}-v_{8}\right),$$

(2) $$M_{y}=F_{x}H-F_{z}D-\frac{Wc}{2}\left(v_{1}+v_{7}-v_{3}-v_{5}\right),\;\mathrm{and}$$

(3) $$F_{z}=c\left(v_{1}+v_{2}+v_{3}+v_{4}-v_{5}-v_{6}-v_{7}-v_{8}\right),$$

where H=15.85 mm, D=5.50 mm, L=3.45 mm, and W=2.95 mm. $c=3.063\;NV^{-1}$ is the voltage change per unit force, and $v_{i}$ the voltage output of the *i*th sensor as labeled in Figure 3. From Equations (1)–(3), we can express the measured forces as

(4) $$\left[\begin{array}{c} F_{x}\\ F_{y}\\ F_{z} \end{array}\right]=A\left[\begin{array}{c} v_{1}\\ \vdots \\ v_{8} \end{array}\right],$$

where $A\in R^{8\times 3}$ is a sensitivity matrix that maps sensor voltage outputs to forces. Using the values of *H*, *L*, *W*, *D*, and *c*, the theoretical value of A is given as

(5) $$A={\left[\begin{array}{ccc} 1.728 & 0 & 3.063\\ 1.062 & -0.570 & 3.063\\ 0.396 & 0 & 3.063\\ 1.062 & 0.570 & 3.063\\ -1.728 & 0 & -3.063\\ -1.062 & -0.570 & -3.063\\ -0.396 & 0 & -3.063\\ -1.062 & 0.570 & -3.063 \end{array}\right]}^{T},$$

where each element of *A* has units of $NV^{-1}$. Using *A*, we can compute the pseudo-inverse,

(6) $$\begin{array}{cc} A^{†} & =A{\left(AA^{T}\right)}^{-1}\\ \; & =\left[\begin{array}{ccc} 0.375 & 0 & -0.089\\ 0 & 0.439 & 0.041\\ -0.375 & 0 & 0.171\\ 0 & -0.439 & 0.041\\ -0.375 & 0 & 0.089\\ 0 & 0.439 & -0.041\\ 0.375 & 0 & -0.171\\ 0 & -0.439 & -0.041 \end{array}\right], \end{array}$$

which provides the least-norm solution, allowing us to predict the theoretical sensor response to variations in forces applied to the sensor,

(7) $$\left[\begin{array}{c} v_{1}\\ \vdots \\ v_{8} \end{array}\right]=A^{†}\left[\begin{array}{c} F_{x}\\ F_{y}\\ F_{z} \end{array}\right].$$

The configuration of the load cells with respect to the sensing plate produces a predicted sensor response in x-, y-, and z-directions of force that is described by $A^{†}$. Sensor 1 and Sensor 7 voltages increase when the sensor is loaded in the positive x-direction while their opposing sensors, 3 and 5, decrease, and vice-versa for the negative x-direction. Sensor 2 and Sensor 6 voltages increase when the sensor is loaded in the positive y-direction while their opposing sensors, 4 and 8, decrease, and vice-versa for the negative y-direction. All sensors respond to loading in the z-direction. Because the loading in the z-axis of the force sensor is offset from the principal axis of the sensing rod, loading in the z-direction produces a moment about the y-axis. Thus, during loading in the positive z-direction, the voltage of Sensors 1 and 7 decrease, while those of their opposing sensors, 3 and 5, increase. Since in pure z-direction loading there is no moment about the x-axis, the voltages at Sensors 2 and 4 increase, while their opposing Sensors 6 and 8 decrease.

### 3.4. Calibration Method

The actual value of *A* can be estimated through linear least-squares fitting to calibration data. To improve the quality of calibration, we also fit a constant offset term for each direction of force. This results in the estimated sensitivity matrix $A^{†}$ having a dimension of 3×9.

**Figure 4:**
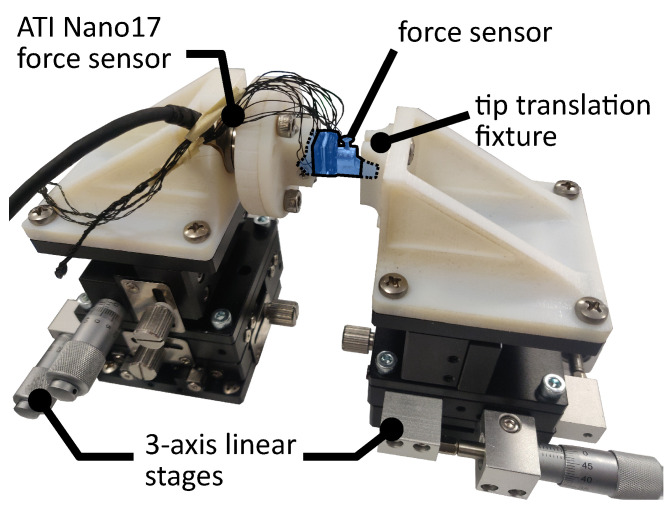
Setup used for static calibration of a single force sensor.

To perform the calibration, the sensor was mounted on a Nano17 force sensor (ATI, Apex, NC, USA). The tip of the jaw attachment was affixed to a 3-axis linear stage as shown in Figure 4 and loaded in each Cartesian axis in increments of 0.5 ± 0.1 N through the target sensing range of 0 to ±3 N in the x- and y-directions and 0 to ±5 N in the z-direction. To reduce calibration errors due to possible hysteretic behavior, data were collected during loading and unloading. The quality of the calibration was evaluated using the root mean square error (RMSE), the normalized root mean square deviation (NRMSD), which is the RMSE normalized by the measurement range of the sensor, the coefficient of determination ($R^{2}$), and the hysteresis, which is the maximum difference between corresponding measured forces during loading and unloading normalized by the maximum force [37].

### 3.5. Performance Evaluation

#### 3.5.1. Single-Jaw Evaluation

The single-jaw evaluation was performed by exerting varying loads on the tip of the jaw attachment in all three Cartesian directions while the sensor was mounted to a Nano17 force sensor using the calibration setup shown in Figure 4 without the tip translation fixture. The sensor accuracy was determined by computing the RMSE and NRMSD of the force measurements and averaging them over three trials. To quantify the worst case error in each sensor axis, the maximum error of the sensor during evaluation was measured. This is defined as the largest error measured in each of the sensor axes over all three evaluation trials. It is expressed as an absolute force value and as a percentage of the range of the sensor.

#### 3.5.2. Dual-Jaw Evaluation

To measure the manipulation forces at the end effector, each jaw of the da Vinci large needle driver needs to be instrumented with a force sensor. The forces measured in the reference frame of each sensor are first resolved into the reference frame of a da Vinci Research Kit (dVRK) [38]. This is done using the robot forward kinematic model and the joint position estimates from the motor encoders (6 joints and the gripper angle) to obtain the individual jaw poses in the robot reference frame. The resultant force, $F_{r}$, is thus

**Figure 5:**
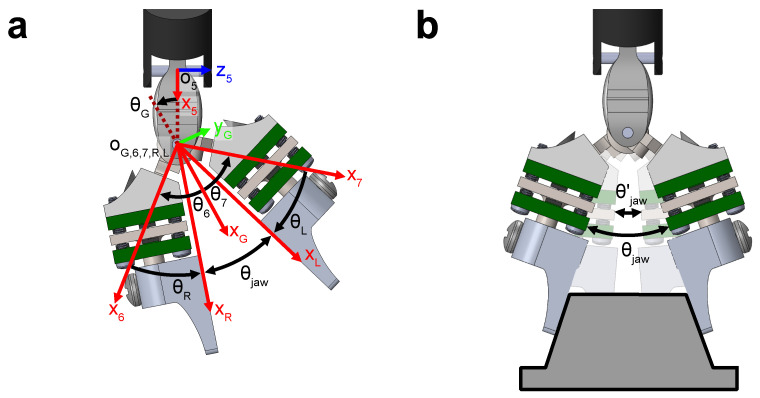
(**a**) Definitions of angles and coordinate frames at the gripper of the sensorized RMIS tool. (**b**) Angle definitions for the software reported jaw angle $\theta_{\mathrm{jaw}}^{{}^{\prime }}$ and the actual jaw angle $\theta_{\mathrm{jaw}}$.

(8) Fr0=T60(θ1,…,θ5,θ6,θG)TR6(θR)FR+T70(θ1,…,θ5,θ7,θG)TL7(θL)FL,

where Tij are transformation matrices describing transformations mapping frame *i* to *j*, with frame 0 being the dVRK origin, frames 6 and 7 describing the left and right tool gripper jaws respectively, and the L and R frames describing the local coordinate frames of each force sensor. The values of $\theta_{0}$ to $\theta_{7}$ are joint rotation angles, $\theta_{R}$ and $\theta_{L}$ are fixed rotation angles, and $\theta_{G}$ is the angle between the x-axis of frame 5 and the bisector of $\theta_{6}$ and $\theta_{7}$ (Figure 5a).

Due to backlash and stretching of the tool actuation tendons, the computed jaw angle $\theta_{\mathrm{jaw}}^{{}^{\prime }}$ during grasping, as derived from the estimates of $\theta_{6}$ and $\theta_{7}$ and values of $\theta_{R}$ and $\theta_{L}$, is smaller than the actual sensorized tool jaw angle $\theta_{\mathrm{jaw}}$ (Figure 5b). To ensure that the correct joint angles are used during the pose computation, we define $\theta_{\mathrm{jaw}}$ such that

(9) $$\theta_{\mathrm{jaw}}=\left(\theta_{6}-\theta_{R}\right)+\left(\theta_{7}-\theta_{L}\right)=\left\{\begin{array}{cc} \theta_{\mathrm{jaw}}^{{}^{\prime }}, & \mathrm{if}\;\theta_{\mathrm{jaw}}^{{}^{\prime }}>\theta_{\min}\\ \theta_{\min}, & \mathrm{otherwise} \end{array}\right.$$

where $\theta_{6}=\theta_{7}$, and $\theta_{\min}$ is the minimum jaw angle during grasp.

Because each jaw is instrumented with a force sensor, the grasp force between the two jaws can be obtained. The grasp force was computed by using a two-point grasp model and resolving the forces measured at each sensor into the line of action between the two grasp points. Applying the rules derived by Yoshikawa and Nagai [39], the grasp force for a two-point grasp is

(10) Fg=min(|(TRGFR)·j^|,|(TLGFL)·j^|),

where *G* denotes the gripper frame of reference as shown in Figure 5a, and $\hat{j}={\left[\begin{array}{ccc} 0 & 1 & 0 \end{array}\right]}^{⊺}$.

**Figure 6:**
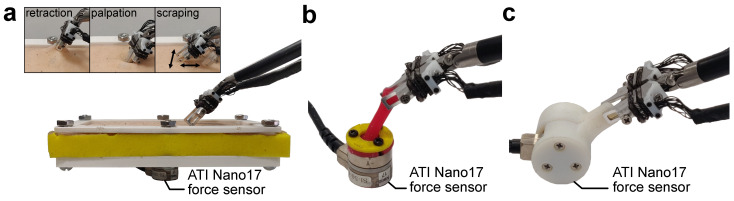
Setups for evaluating performance of force sensors (shown here with strain relief brackets) when mounted on the da Vinci large needle driver tool. (**a**) The flat tissue manipulation (with inset showing, from left to right, retraction, palpation and scraping movements), (**b**) the cylindrical stem manipulation, and (**c**) the grip force measurement setups.

To evaluate the sensor on realistic tissue manipulation tasks, we designed two environments that enable different types of manipulation forces to be exerted by an instrumented RMIS tool mounted on a teleoperated dVRK. As shown in Figure 6a, the first environment consisted of artificial silicone tissue (Limbs and Things, Savannah, GA, USA) placed over a sponge. The artificial tissue and sponge were then clamped down to a rigid stage using a plastic flange. A Nano17 force sensor was placed underneath the stage, such that the stage was fully supported by the sensing element of the Nano17. Thus, all tool–tissue interaction forces are transmitted directly to the Nano17. In this environment, the tool can be teleoperated to perform palpation, scraping, and tissue retraction. However, due to the low friction of the silicone as well as the need to limit grasp forces in software to protect the sensor from damage, the tissue retraction force achievable in this environment was low compared to the sensor’s operating range. The second environment consisted of a cylindrical silicone stem mounted on top of a Nano17 force sensor (Figure 6b). This setup thus allowed the teleoperator to exert higher retraction forces on the environment. In these two setups, the ground truth force during the teleoperated interactions could be obtained and used to evaluate the RMSE, RMSD, and maximum error of the resultant force was measured from the dual-jaw sensors over three trials of each task. However, the grip forces cannot be evaluated.

To evaluate grip force, a separate experiment was devised. This involved attaching 3D-printed cantilevers to each interface of the Nano17 force sensor, leaving the sensor ungrounded, as shown in Figure 6c. The sensorized RMIS tool was then teleoperated to grasp and release the opposing cantilevers five times each in three different trials. The sensor accuracy was determined by computing the RMSE and normalized root mean square deviation of the force measurements and averaging them over three trials. The maximum error over three trials was also calculated.

## 4. Results and Discussion

### 4.1. Static Calibration

As described in Section 3.5.2, sensing of manipulation and grip forces at the end effector required two sensors to be fabricated. Thus, the calibration was performed on two sensors, A and B, each corresponding to one jaw. The estimated sensitivity matrix for sensor A was

(11) $$A_{A}^{†}={\left[\begin{array}{ccc} 1.685 & 0.084 & 3.016\\ 0.940 & 0.663 & 3.148\\ 0.140 & 0.099 & 3.049\\ 1.088 & -0.622 & 3.099\\ -1.795 & 0.0274 & -3.158\\ -0.895 & 0.569 & -3.241\\ -0.349 & 0.010 & -3.191\\ -0.898 & -0.866 & -3.016\\ 0.029 & 0.001 & 0.004 \end{array}\right]}^{T},$$

while for sensor B it was

(12) $$A_{B}^{+}={\left[\begin{array}{ccc} 1.506 & 0.195 & 2.563\\ 1.216 & 0.723 & 3.107\\ 0.488 & 0.016 & 3.114\\ 1.114 & -0.474 & 3.108\\ -1.931 & -0.016 & -3.084\\ -1.104 & 0.572 & -3.107\\ -0.231 & -0.153 & -2.342\\ -1.064 & -0.950 & -3.136\\ 0.045 & 0.073 & 0.020 \end{array}\right]}^{T}.$$

**Figure 7:**
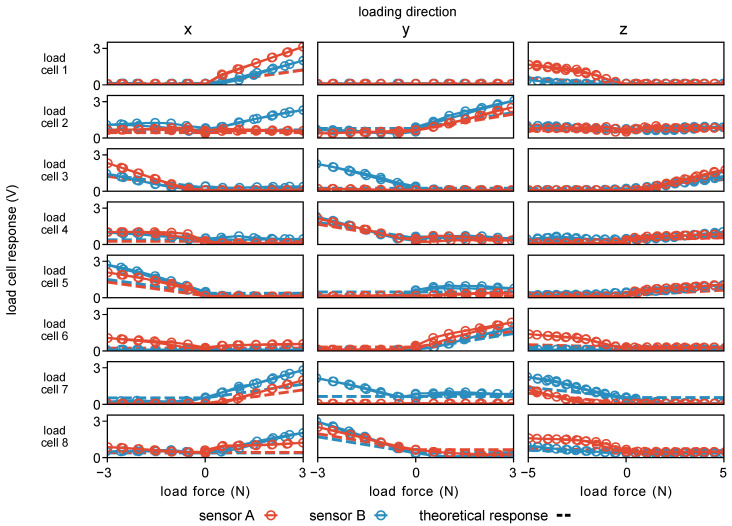
Load cell (LC) response under loading in each Cartesian direction. Load forces were measured using an ATI Nano17 force sensor within the calibration setup shown in Figure 4.

**Figure 8:**
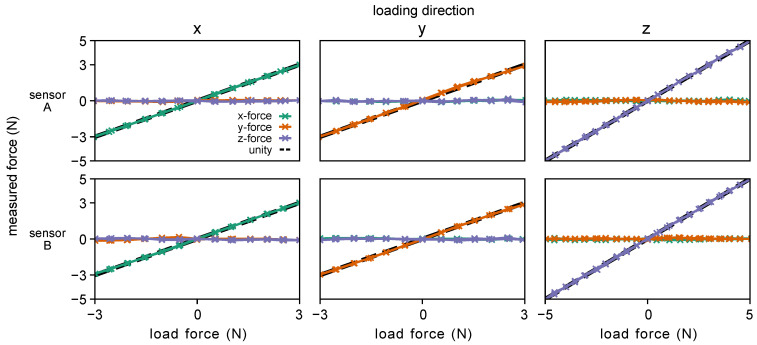
Forces measured by the force sensors in each axis when loaded and unloaded independently in each Cartesian direction.

The load cell responses during loading in each Cartesian direction during calibration are shown in Figure 7 and indicate that, when the sensing principle described in Section 3.3 predicted a response (dashed lines) from a given load cell, there was an appropriate response from that corresponding load cell. Additionally, there were some unexpected responses when no response was predicted due to sensor cross-talk and uneven plate contact that arose from slight errors in manufacturing. Overall, the sensors displayed good linearity over their functional range (Figure 8), with the redundant sensing architecture of the sensor mitigating any detrimental effects of crosstalk on sensor performance. The low deviation from unity in both loading and unloading seen in Figure 8 also indicates that the sensor has low hysteresis despite its non-monolithic design. The results of the calibration procedure are summarized in Table 1.

### 4.2. Single-Jaw Evaluation

**Figure 9:**
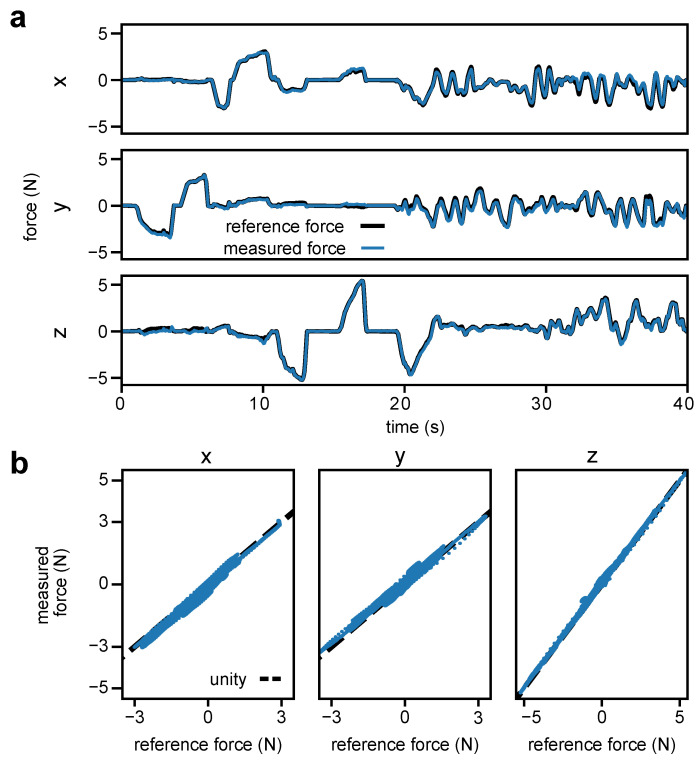
Selected results from single-jaw evaluation of sensor B. (**a**) Recorded forces over time. (**b**) Measured force from the force sensor versus the reference force in each of the three Cartesian directions.

The results of the single-jaw evaluation are summarized in Table 2. The low RMSEs (up to 0.146 N for sensor B in the y-direction) showed that the sensor performance seen in calibration translated to a good performance in the dynamic loading scenario of the single-jaw evaluation. As seen in Figure 9a, from 25 s to 40 s, the sensor could accurately track fast changes in applied force while maintaining the desired accuracy of below 0.375 N RMSE.

The sensor showed a maximum error of 0.654 N for sensor B in the y-direction. The maximum errors occur when the device is loaded in multiple directions, with the z-direction being loaded above 2.2 N. For sensor B, the maximum force error of 0.573 N in the x-direction was measured when −0.844 N, −0.062 N, and 2.19 N of force was exerted in the x-, y-, and z-directions, respectively. The y-direction maximum force error of 0.654 N was measured when the force exerted was −1.27 N, 0.245 N, and −4.94 N in the x-, y-, and z-directions, respectively. The z-direction maximum force error of 0.536 N was measured when the force exerted was 0.353 N, −0.223 N, and −2.537 N in the x-, y-, and z-directions, respectively. The observed inaccuracies are due to the use of a load cell array for sensing, as opposed to a single unified sensing element. The moderate loading in the z-direction reduces the contact force of the plate with the load cell sensing elements. This can cause the sensing plate to shift within the mechanical tolerances of the assembly when combined with a lateral load, resulting in the observed deviations from the static calibration. Thus, the measured versus reference force plot in Figure 9b shows some deviation from unity for all three Cartesian directions.

### 4.3. Dual-Jaw Evaluation

**Figure 10:**
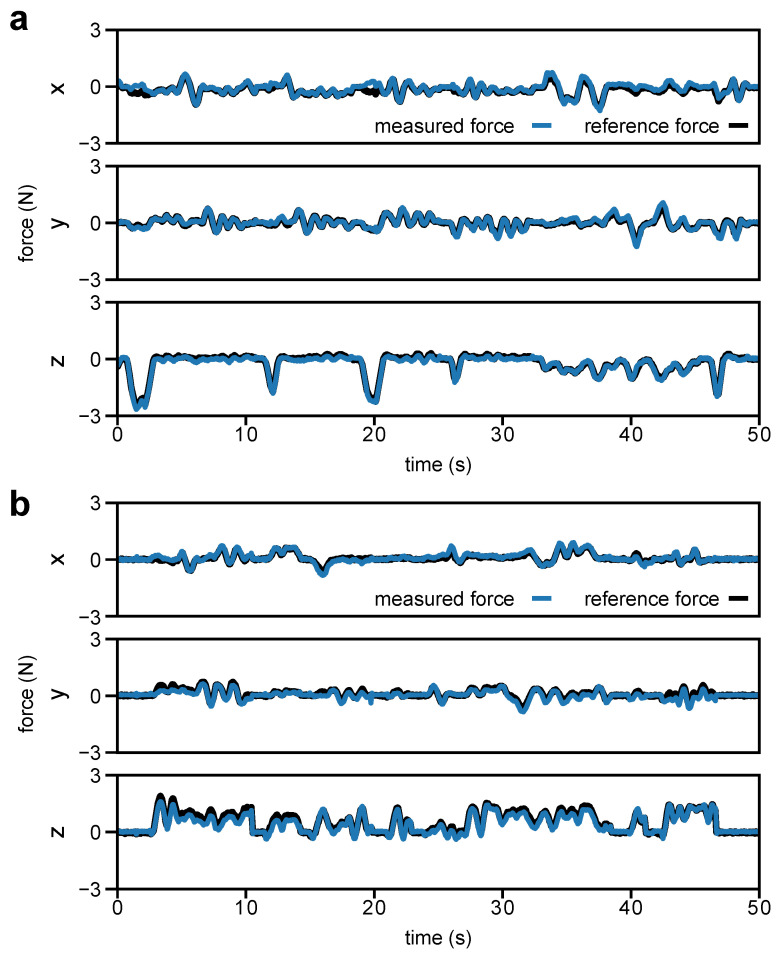
Selected results for the (**a**) flat tissue and (**b**) cylindrical stem manipulation tasks.

For both of the dual-jaw evaluation tasks, we used a minimum jaw angle of $\theta_{\min}=8.4^{\circ }$. This was required because the gripper does not fully close during grasping; thus, the dVRK would report an incorrect gripper pose. This minimum jaw angle was empirically determined to reduce the error in the x-direction force measurements of both tasks with the RMSE of the sensor being below the minimum target of 0.375 N, as summarized in Table 3. The force measurements from the sensor with respect to the ground truth for selected trials of the flat tissue and cylindrical stem manipulation tasks are shown in Figure 10. The plots show good tracking performance for lateral forces (x- and y-direction in Figure 10a,b), as well as for palpation (z-direction in Figure 10a) and tension (z-direction in Figure 10b). A video showing examples for each of the dual jaw evaluation tasks is provided in the Supplementary Materials (Video S1). The maximum error in the x-direction of the flat tissue task is 0.725 N, as shown in Table 3. This maximum error was measured when the resultant force applied to the artificial tissue was −0.354 N, −0.499 N, and −2.808 N in the x-, y-, and z-directions of the da Vinci reference frame, respectively. In this configuration, the local z-axes of the sensors are aligned with the z-direction of the da Vinci reference frame. Thus, in the sensor reference frame, there is cross-loading of lateral forces with a primary axial (sensor z-direction) component of force similar to the scenario described in Section 4.2. This contributed to a larger error compared to the other occurrences of maximum error shown in Table 3.

In addition to the sources of error from the single jaw, we identified three extrinsic sources of error. First is the pose uncertainty of the jaws during grasping due to the cable-driven design of the dVRK robot, where encoders are not placed directly on each joint of the surgical tool. Second is the varying point of force application on the sensor, in which its calibration was accomplished with forces applied only to the tip of the jaw attachment (Figure 3 and Figure 4). Third is the small misalignment between the robot base coordinate frame and the reference force sensor coordinate frame.

### 4.4. Grip Force Evaluation

**Figure 11:**
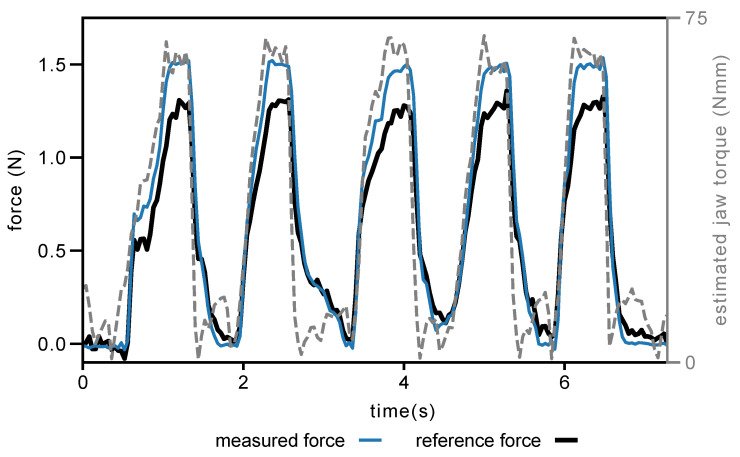
Selected grip force measurement results. Estimated jaw torque is derived from the motor current measurements at the instrument base as reported through the dVRK API.

For the grip force evaluation, the RMSEs and standard deviations over three trials were 0.156 ± 0.017 N, and the maximum errors were 0.287 N (19.79% of the peak grip force). A sample force plot over time using the default minimum jaw angle is shown in Figure 11. A video of an example of the grip force evaluation task is provided in the Supplementary Materials (Video S1).

The dVRK requires the teleoperator to momentarily close the jaws to trigger teleoperation, this movement causes the tool jaws to snap together, resulting in an impulsive load on the sensors. To prevent damage to the sensors during this movement, we limited the maximum grip force in software. Because of this limit, the highest peak grip force achieved during the evaluation was 1.45 N, and the average peak grip force was 1.35 N, which is below the range of our sensor. The limit also prevented us from evaluating the dual-jaw performance of the sensor in both manipulation tasks up to the same range used in single-jaw evaluation. The sample would slip from grasp before the higher forces were reached. With modification of the underlying dVRK teleoperation code, it would be possible for the sensors to be tested to their lateral force upper range of 3 N.

## 5. Conclusions

In this work, we presented a 3DoF force sensor designed to facilitate RMIS research. The sensor can be used with an existing RMIS tool, is manufacturable using low-volume manufacturing techniques, and has interchangeable jaws that allow for adaptation to different RMIS tasks.

The current design is tolerant to manufacturing variability, leading to robust performance. In both single-jaw standalone evaluation and dual-jaw evaluation on an RMIS tool, the sensor met the target accuracy specification of less than 0.375 N RMSE. In single-jaw evaluation, this accuracy was verified within the target sensing range of ±3 N for the lateral (x and y) directions and ±5 for the axial (z) direction of force.

Future work will investigate approaches to expand the sensor range. We will also investigate approaches to mitigate the uncertainty that arises from cross-talk between load cells. One promising approach is to reduce the number of load cells to three per array, which would produce a statically determinate design that comes at the expense of reduced sensing range. This approach also reduces the reliance on manual shimming of the sensor assembly components. To reduce the shifting of the plate, which contributes to high maximum errors of up to 11% under cross-loading, we will develop deformable inserts that can be placed in the gaps between the load cell arrays and the sensing plate.

In the dual-jaw evaluation, the chief contributor to error was the pose uncertainty of the tool wrist and jaws. The uncertainty was most pronounced during high force manipulations and in grasping. To enable consistent and maximally accurate force measurements in cable-driven RMIS platforms such as the dVRK, pose measurement approaches such as those based on stereovision [40,41] or robot state information [42,43,44] will need to be further developed to improve real-time accuracy. Even with the limitation in pose measurement accuracy, the dual jaw sensor meets the human perception-based performance specifications and is a promising tool for enabling RMIS research that requires force information for bimanual tasks. The sensor designs have been made freely available online to allow researchers to manufacture and modify the sensor for use in applications such as providing haptic feedback, performing robotic force control, and collecting bimanual RMIS manipulation datasets that inform data-driven computational methods. 

## Figures and Tables

**Table 1:** Summary of static calibration results for both sensors.

Sensor	RMSE (N)			NRMSD (%)			$R^{2}$			Hysteresis (%)		
	x	y	z	x	y	z	x	y	z	x+, x−	x+, x−	z+, z−
A	0.023	0.056	0.044	0.388	0.876	0.438	0.999	0.996	0.999	3.96, 3.48	3.25, 2.85	2.78, 2.37
B	0.032	0.048	0.045	0.531	0.814	0.445	0.999	0.997	0.999	2.13, 2.73	2.15, 1.62	3.17, 3.36

**Table 2:** Summary of single-jaw evaluation results for both sensors.

Sensor	RMSE (N)			NRMSD (%) *			Max Error (N) *		
	x	y	z	x	y	z	x	y	z
A	0.111 ± 0.016	0.105 ± 0.015	0.064 ± 0.004	1.845 ± 0.278	1.745 ± 0.246	0.635 ± 0.036	0.483 (8.05%)	0.415 (6.76%)	0.325 (3.24%)
B	0.117 ± 019	0.146 ± 0.013	0.126 ± 0.012	1.945 ± 0.316	2.43 ± 0.220	1.264 ± 0.123	0.573 (9.55%)	0.654 (10.90%)	0.536 (5.36%)

* Normalized over the range of forces in evaluation, which are ±3 N in x and y and ±5 N in z.

**Table 3:** Summary of dual-jaw evaluation results for the flat tissue and cylindrical stem manipulation tasks.

Task	RMSE (N)			NRMSD (%)			Max Error (N) *		
	x	y	z	x	y	z	x	y	z
Flat tissue	0.142 ± 0.020	0.078 ± 0.013	0.097 ± 0.008	2.367 ± 0.338	1.300 ± 0.225	0.980 ± 0.082	0.725 (12.09%)	0.452 (7.54%)	0.458 (4.85%)
Cylindrical stem	0.089 ± 0.020	0.149 ± 0.023	0.139 ± 0.012	1.485 ± 0.327	2.481 ± 0.389	1.392 ± 0.117	0.357 (5.94%)	0.484 (8.07%)	0.458 (7.64%)

* Normalized over the range of forces in evaluation, which are ±3 N in x and y and ±5 N in z of the da Vinci reference frame.

## Data Availability

The sensor mechanical and electrical design files, microprocessor code, and processing scripts can be accessed at the following GitHub repository: https://github.com/enhanced-telerobotics/RMIS_force_sensor/ (accessed on 25 May 2023).

## Supplementary Materials

The following supporting information can be downloaded at: https://www.mdpi.com/article/10.3390/s23115230/s1, Video S1: Demonstration of force sensors for dual-jaw manipulation. Video S2: Assembly instructions for a single-sensor assembly.

## Abbreviations

The following abbreviations are used in this manuscript:

RMIS	Robot-assisted Minimally Invasive Surgery
DoF	Degrees-of-Freedom
IR	Infrared Radiation
